# The genomic signature of trait-associated variants

**DOI:** 10.1186/1471-2164-14-108

**Published:** 2013-02-18

**Authors:** Alida S D Kindt, Pau Navarro, Colin A M Semple, Chris S Haley

**Affiliations:** 1 MRC Human Genetics Unit, MRC Institute of Genetics and Molecular Medicine, University of Edinburgh, Western General Hospital, Crewe Road, EH4 2XU, Edinburgh, UK

**Keywords:** GWAS, Trait-associated SNPs, Chromatin states, Genomic features, Permutations, Logistic regression, Prioritization

## Abstract

**Background:**

Genome-wide association studies have identified thousands of SNP variants associated with hundreds of phenotypes. For most associations the causal variants and the molecular mechanisms underlying pathogenesis remain unknown. Exploration of the underlying functional annotations of trait-associated loci has thrown some light on their potential roles in pathogenesis. However, there are some shortcomings of the methods used to date, which may undermine efforts to prioritize variants for further analyses. Here, we introduce and apply novel methods to rigorously identify annotation classes showing enrichment or depletion of trait-associated variants taking into account the underlying associations due to co-location of different functional annotations and linkage disequilibrium.

**Results:**

We assessed enrichment and depletion of variants in publicly available annotation classes such as genic regions, regulatory features, measures of conservation, and patterns of histone modifications. We used logistic regression to build a multivariate model that identified the most influential functional annotations for trait-association status of genome-wide significant variants. SNPs associated with all of the enriched annotations were 8 times more likely to be trait-associated variants than SNPs annotated with none of them. Annotations associated with chromatin state together with prior knowledge of the existence of a local expression QTL (eQTL) were the most important factors in the final logistic regression model. Surprisingly, despite the widespread use of evolutionary conservation to prioritize variants for study we find only modest enrichment of trait-associated SNPs in conserved regions.

**Conclusion:**

We established odds ratios of functional annotations that are more likely to contain significantly trait-associated SNPs, for the purpose of prioritizing GWAS hits for further studies. Additionally, we estimated the relative and combined influence of the different genomic annotations, which may facilitate future prioritization methods by adding substantial information.

## Background

Genome-wide association studies (GWAS) have been successful in discovering associated variants for a wide range of common diseases and traits [1,2]. More than 8,000 trait associations have been recorded to date (as of 11 Jan 2013). They can be divided into significant associations passing the generally accepted genome-wide threshold (P-value = 5 × 10^-8^) [3], or suggestive associations with a decreased significance threshold (P-value = 5 × 10^-5^ – 5 × 10^-8^). Larger association studies and increasingly informative genotyping arrays together with high-throughput sequencing are expected to confirm some of the associations currently considered as suggestive and identify many new associations [4]. For confirmed associations, experiments identifying the causal underlying biology are expensive in both time and money. This causes a bottleneck in elucidating the molecular processes and pathways underlying these associations [5-8] and hence in gaining new biological knowledge. There has therefore been much interest in computational prioritization of candidate variants, both to accelerate the search for causal variants, and to provide insights into the biology underlying disease states.

Although confirmed trait-associated SNP will most often not be the causal variants, the surrounding genomic regions in linkage disequilibrium (LD) with associated SNPs are expected to contain causal variants with biological function. While it is clear that trait-associated SNPs are enriched in genic regions the majority of trait-associated variants are not within genes [7,9]. Two studies have previously investigated trait-associated SNP enrichment in a range of genomic features [8,10]. Hindorff et al. (2009) investigated 20 genomic features for enrichment or depletion of trait-associated SNPs [8]. They found non-synonymous sites and 5 Kb regions upstream of transcription start sites significantly enriched for trait-associated SNPs, while intergenic regions were significantly depleted [8]. Knight et al. (2011) [10] replicated the significant enrichment results of Hindorff et al. [8] and additionally found that *cis* expression quantitative trait loci (*cis*-eQTLs) were enriched for associated variants. Studies focusing on the epigenomic landscape, such as DNA methylation [11] and histone modification patterns [12] surrounding the variants have shown SNPs and DNA modifications jointly influencing transcription of nearby genes. Recently, combinatorial patterns of histone modifications were found to indicate regions with particular functions, ranging from active promoters or enhancers to transcriptionally silenced loci [13]. Ernst et al. [13] also showed that GWAS variants associated with diseases showing lineage-specific phenotypes were enriched in enhancer regions predicted using chromatin data from similar cells, e.g. acute lymphoblastic leukemia variants were enriched within strong enhancers found in T cells.

Alternative approaches to functional enrichment analyses have the potential to provide additional insight into the influence of genomic features on trait associations. To study enrichment or depletion both Knight et al. [10] and Hindorff et al. [8] compared the annotations of associated variants to background sets of SNPs present on the original GWAS genotyping platforms. Hindorff et al. [8] generated 100 randomly sampled background SNP sets, weighted to approximate the composition of the genotyping platforms originally used to uncover the associations. Knight et al. [10] calculated enrichments based upon backgrounds composed of all SNPs from two popular genotyping platforms (the Affymetrix 500 K platform, the Illumina HumanHap 550 K platform, and the union of these two platforms). These approaches have important caveats. Firstly, the platform, or combination of platforms, used to detect an association is not always recorded, as shown by the GWAS catalogue [5]. Secondly, the underlying distributions of functional genomics features and SNPs are ignored, although it is known that their distributions in the human genome are often non-uniform and clustered [14]. Sampling randomly selected SNPs implicitly assumes that SNPs occur uniformly across the genome, which may result in misleading conclusions. It is also unclear what level of sampling is sufficient to produce an appropriate null distribution for a given set of variants. If we aim to assess the significance of the co-occurrence of associated SNPs and genomic features, an appropriate background SNP set should reflect the degree to which SNPs and genomic features are clustered and occur in the genome. Finally, previous studies have failed to take account of the often strong inter-dependencies between different genomic features e.g. the associations between chromatin structure, gene density and evolutionary divergence rates [15]. These inter-dependencies make it difficult to disentangle the relative importance of individual genomic features when analyzed separately.

Here, we investigated the genomic signature of 1,909 significantly trait-associated SNPs (P-value < 5 × 10^-8^) by analyzing the overlaps between regions annotated for 58 genomic features with the associated variants and their LD SNP partners. We used a novel circular permutation approach to assess the significance of the observed results and to calculate enrichment or depletion scores for each genomic feature. Our permutation approach preserves the observed distribution of annotations and SNPs around the genome, and establishes a robust null distribution from which the significance of the observed enrichments and depletions can be calculated. We compared the permutation results with results obtained by a sampling strategy based on Hindorff et al. [8], which randomly samples genotyping platforms and SNPs from the HapMap II project present in CEU (Caucasian population). In addition to examining the annotations investigated by Hindorff et al. [8] and Knight et al. [10], we included 15 different annotations relating to chromatin states associated with regulatory regions [13], eQTLs [16], higher order chromatin structure [17] and regions with identified evolutionary signatures [18]. Most of the annotations we examined are correlated with at least some of the others, prompting us to investigate their combined effects. We applied stepwise logistic regression in order to derive a minimum set of enriched or depleted annotations that jointly influence trait-association status. The logistic regression approach accounts for any redundant information carried by individual variables, for example due to co-location of different functional annotations. Additional annotations are only included if they add information that is not explained by other annotations that are already in the model resulting in a final model that incorporates the most important variables only. All analyses performed took the underlying LD structure into account, as all analyzed SNPs – trait-associated and non-associated – were investigated with their LD partners at the chosen LD cut-off. The enrichment/depletion and logistic regression analyses were repeated with another SNP set consisting of 2,410 suggestively trait-associated SNPs (P-value between 5 × 10^-5^ and 5 × 10^-8^). The results shed new light on the genomic architecture of trait-associated SNPs and may be useful to aid prioritization of associated variants for further study and as prior weighting for association studies.

## Results

### Confirmation of functional enrichments by two independent methods

**Figure 1:**
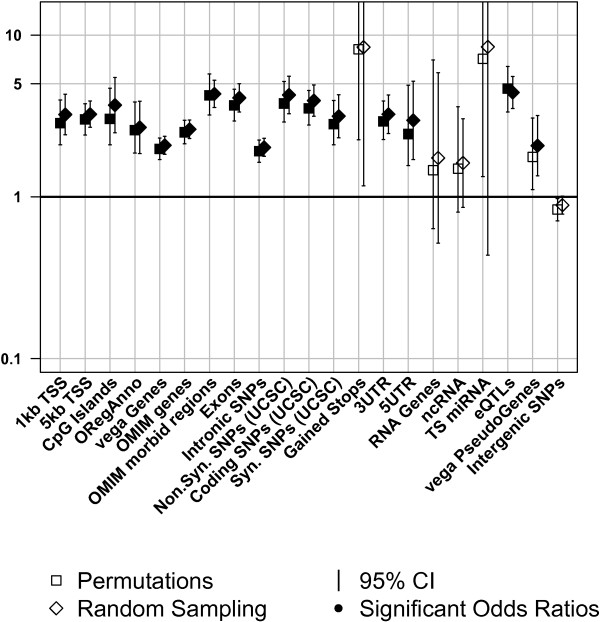
**Genic and regulatory features.** Enrichment of trait-associated SNPs in selected genic features. Sampling and permutation based results are compared (□,◊) for a variety of genic features (see Methods for full details); solid symbols indicate odds ratios significantly different from 1 (p ≤ 0.05). Odds ratios below or above one show depletions or enrichments respectively.

**Figure 2:**
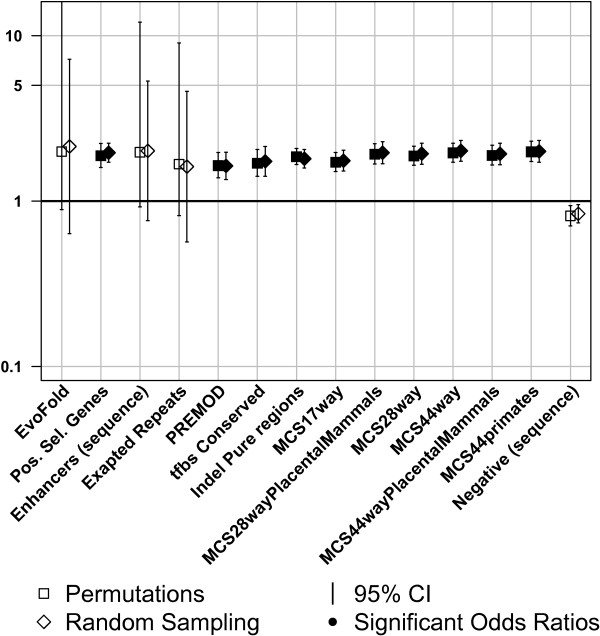
**Regions with conserved and evolutionary signatures.** Enrichment of trait-associated SNPs in selected evolutionary signatures. Sampling and permutation results (□,◊) are compared for regions identified as unusually conserved or divergent by a variety of measures (see Methods for full details); solid symbols indicate odds ratios significantly different from 1 (p ≤ 0.05). Odds ratios below or above one show depletions or enrichments respectively.

**Figure 3:**
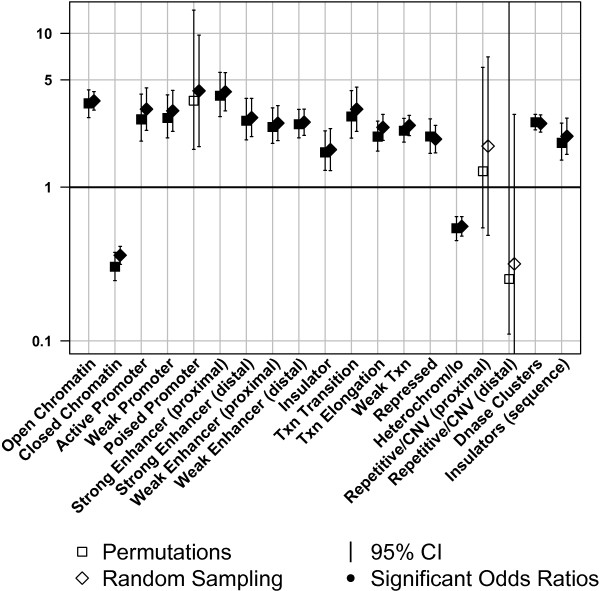
**Chromatin states.** Enrichment of trait-associated SNPs in selected chromatin features. Sampling and permutation based results are compared (□,◊) for regions associated with chromatin states with varying functions (see Methods for full details); solid symbols indicate odds ratios significantly different from 1 (p ≤ 0.05). Odds ratios below or above one show depletions or enrichments respectively.

Figures 1, 2, and 3 display the results obtained by circular permutations and the sampling method for 54 annotations and are summarized according to annotation class. Four of the annotations were excluded from further analyses, as their coverage by all analyzed SNPs was very low, and thus not informative. Summary statistics for the analyzed annotations including the number of observed sites, the percentage of total nucleotides covered in the genome, the percentage of SNPs covered in the genome, and the average length of the annotated sites in base pairs were calculated for all annotations (Additional file 1). Odds ratios, which indicate enrichment/depletion of trait-associated SNPs, were calculated for each annotation. An odds ratio equal to unity indicated that trait-associated SNPs were as likely to coincide with the analyzed genomic feature as non-associated SNPs. An odds ratio above unity indicated that the genomic feature was enriched for trait-associated SNPs, while odds ratios below unity were evidence for depletion. Fold enrichment and odds ratios were approximately equivalent, (see Additional file 2 and Additional file 3). In general, the odds ratios for a particular annotation were very similar between the sampling and permutation approaches with odds ratios correlating strongly (r^2^ = 0.98, P-value = 1.01 × 10^-51^). However, two (vega PseudoGenes and inactive/poised promoters) of 54 annotations obtained different significance using the two different methods. The odds ratios associated with these two annotations are almost identical, which means that the differences are due to different confidence intervals obtained by the two methods. Table 1 shows a comparison of odds ratios and P-values obtained using the permutation and sampling methods on the significantly and suggestively trait-associated SNPs. Table 2 shows the average of the CI widths for each of the three annotation classes per method. Note that P-values from permutation were truncated at <5.00 × 10^-5^ due to the number of permutations performed (i.e. 20,000). A more extreme threshold would not materially change the conclusions and each order of magnitude decrease in the threshold requires an order of magnitude increase in the number of permutations and hence computation. The effect of the annotation was declared significant if the observed P-value passed the significance threshold set by the Bonferroni correction for the number of annotations studied (P-value ≤ 8.62 × 10^-4^).

**Table 1:** Odds ratios of sampling and permutations for two datasets

	**Sampling**		**Permutations**			
	**Significantly associated SNPs**		**Significantly associated SNPs**		**Suggestively associated SNPs**	
	**OR [95% CI]**	**P-value**	**OR [95% CI]**	**P-value**	**OR [95% CI]**	**P-value**
		**Genic and regulatory features**				
TSS 1 kb upstream	3.23 [2.42-4.31]	3.25 × 10 ^-17^	2.86 [2.10-3.97]	<5.00 × 10 ^-05^	1.25 [1.01-1.60]	1.94 × 10 ^-02^
TSS 5 kb upstream	3.24 [2.69-3.91]	1.56 × 10 ^-38^	3.01 [2.41-3.76]	<5.00 × 10 ^-05^	1.24 [1.08-1.44]	9.00 × 10 ^-04^
CpG Islands	3.69 [2.49-5.47]	2.09 × 10 ^-12^	3.03 [2.10-4.70]	<5.00 × 10 ^-05^	1.05 [0.79-1.49]	4.05 × 10 ^-01^
ORegAnno	2.69 [1.85-3.90]	6.24 × 10 ^-08^	2.58 [1.86-3.85]	<5.00 × 10 ^-05^	1.37 [1.05-1.91]	8.40 × 10 ^-03^
vega Genes	2.08 [1.83-2.37]	1.84 × 10 ^-29^	1.98 [1.70-2.31]	<5.00 × 10 ^-05^	1.17 [1.08-1.28]	5.00 × 10 ^-05^
OMIM Genes	2.62 [2.30-2.98]	1.35 × 10 ^-48^	2.51 [2.13-2.97]	<5.00 × 10 ^-05^	1.24 [1.13-1.35]	<5.00 × 10 ^-05^
OMIM Morbid Regions	4.33 [3.57-5.25]	5.95 × 10 ^-58^	4.24 [3.20-5.75]	<5.00 × 10 ^-05^	1.38 [1.18-1.62]	<5.00 × 10 ^-05^
Exons	4.09 [3.34-5.01]	5.35 × 10 ^-49^	3.67 [2.94-4.62]	<5.00 × 10 ^-05^	1.39 [1.20-1.65]	<5.00 × 10 ^-05^
Intronic SNPs	2.02 [1.78-2.30]	1.92 × 10 ^-27^	1.92 [1.63-2.25]	<5.00 × 10 ^-05^	1.15 [1.06-1.26]	3.00 × 10 ^-05^
Non-Syn. SNPs	4.26 [3.26-5.57]	2.24 × 10 ^-31^	3.78 [2.90-5.17]	<5.00 × 10 ^-05^	1.37 [1.12-1.70]	7.00 × 10 ^-04^
Coding SNPs	3.94 [3.15-4.92]	1.73 × 10 ^-38^	3.52 [2.78-4.55]	<5.00 × 10 ^-05^	1.37 [1.16-1.63]	5.00 × 10 ^-05^
Syn. SNPs	3.15 [2.32-4.28]	6.45 × 10 ^-15^	2.81 [2.10-3.94]	<5.00 × 10 ^-05^	1.24 [1.00-1.59]	3.09 × 10 ^-02^
Gained Stops	8.42 [1.17-60.53]	1.04 × 10 ^-02^	8.17 [2.26-Infinity]	9.50 × 10 ^-04^	4.74 [1.50-Infinity]	2.10 × 10 ^-03^
3′UTR	3.24 [2.46-4.26]	2.69 × 10 ^-19^	2.92 [2.26-3.92]	<5.00 × 10 ^-05^	1.29 [1.07-1.61]	4.75 × 10 ^-03^
5′UTR	2.97 [1.70-5.19]	5.08 × 10 ^-05^	2.45 [1.56-4.90]	5.00 × 10 ^-05^	1.37 [0.97-2.30]	5.71 × 10 ^-02^
RNA Genes	1.74 [0.52-5.85]	3.80 × 10 ^-01^	1.46 [0.64-7.02]	2.25 × 10 ^-01^	0.50 [0.27-1.50]	6.21 × 10 ^-02^
ncRNA	1.62 [0.86-3.04]	1.57 × 10 ^-01^	1.49 [0.80-3.61]	1.08 × 10 ^-01^	1.38 [0.93-2.35]	6.73 × 10 ^-02^
tSmiRNA	8.46 [0.44-164.37]	5.83 × 10 ^-02^	7.13 [1.33-Infinity]	9.95 × 10 ^-03^	0.00 [0.00-Infinity]	<5.00 × 10 ^-05^
eQTLs	4.42 [3.52-5.54]	7.12 × 10 ^-45^	4.67 [3.35-6.39]	<5.00 × 10 ^-05^	1.94 [1.61-2.40]	<5.00 × 10 ^-05^
vega PseudoGenes	2.07 [1.35-3.19]	8.09 × 10 ^-04^	1.76 [1.11-3.07]	1.16 × 10 ^-02^	1.10 [0.82-1.58]	2.85 × 10 ^-01^
Intergenic SNPs	0.89 [0.78-1.01]	7.19 × 10 ^-02^	0.83 [0.71-0.98]	1.11 × 10 ^-02^	0.94 [0.86-1.03]	8.68 × 10 ^-02^
		**Conserved regions and evolutionary signatures**				
Evofold	2.14 [0.63-7.20]	2.59 × 10 ^-01^	1.99 [0.89-Infinity]	7.47 × 10 ^-02^	0.79 [0.40-4.00]	2.60 × 10 ^-01^
Pos. Sel. Genes	1.96 [1.71-2.24]	2.05 × 10 ^-23^	1.88 [1.60-2.23]	<5.00 × 10 ^-05^	1.12 [1.03-1.23]	6.65 × 10 ^-03^
Enhancers	2.01 [0.76-5.32]	1.60 × 10 ^-01^	1.97 [0.92-12.07]	5.17 × 10 ^-02^	1.02 [0.57-2.67]	4.72 × 10 ^-01^
Exapted Repeats	1.62 [0.57-4.60]	4.47 × 10 ^-01^	1.67 [0.82-9.04]	1.23 × 10 ^-01^	0.88 [0.46-3.00]	3.25 × 10 ^-01^
PREMOD	1.63 [1.35-1.97]	4.22 × 10 ^-07^	1.64 [1.38-1.97]	<5.00 × 10 ^-05^	1.07 [0.94-1.22]	1.78 × 10 ^-01^
tfbsConsSites	1.74 [1.41-2.14]	1.64 × 10 ^-07^	1.69 [1.41-2.05]	<5.00 × 10 ^-05^	1.02 [0.89-1.18]	4.23 × 10 ^-01^
InDels	1.80 [1.58-2.05]	3.61 × 10 ^-19^	1.85 [1.66-2.08]	<5.00 × 10 ^-05^	1.11 [1.02-1.21]	7.15 × 10 ^-03^
MCS17way	1.75 [1.52-2.03]	1.61 × 10 ^-14^	1.72 [1.51-1.97]	<5.00 × 10 ^-05^	1.01 [0.91-1.11]	4.62 × 10 ^-01^
MCS28wayPlacMammal	1.96 [1.68-2.28]	6.51 × 10 ^-18^	1.92 [1.67-2.22]	<5.00 × 10 ^-05^	1.03 [0.93-1.15]	3.05 × 10 ^-01^
MCS28way	1.93 [1.67-2.24]	6.32 × 10 ^-19^	1.87 [1.65-2.15]	<5.00 × 10 ^-05^	1.00 [0.91-1.11]	4.99 × 10 ^-01^
MCS44way	2.01 [1.74-2.33]	2.10 × 10 ^-21^	1.96 [1.72-2.24]	<5.00 × 10 ^-05^	1.02 [0.93-1.13]	3.33 × 10 ^-01^
MCS44wayPlacental	1.93 [1.66-2.24]	5.26 × 10 ^-18^	1.89 [1.65-2.17]	<5.00 × 10 ^-05^	1.01 [0.92-1.13]	4.10 × 10 ^-01^
MCS44wayPrimates	2.00 [1.72-2.33]	1.42 × 10 ^-19^	1.98 [1.73-2.30]	<5.00 × 10 ^-05^	1.00 [0.91-1.12]	4.69 × 10 ^-01^
Sequence annotation negative	0.84 [0.74-0.95]	6.34 × 10 ^-03^	0.81 [0.71-0.94]	1.95 × 10 ^-03^	0.91 [0.84-0.99]	1.53 × 10 ^-02^
		**Chromatin signatures**				
Open Chromatin	3.65 [3.18-4.18]	2.73 × 10 ^-80^	3.51 [2.84-4.30]	<5.00 × 10 ^-05^	1.31 [1.19-1.44]	<5.00 × 10 ^-05^
Closed Chromatin	0.36 [0.31-0.41]	1.22 × 10 ^-51^	0.30 [0.25-0.38]	<5.00 × 10 ^-05^	0.77 [0.70-0.84]	<5.00 × 10 ^-05^
Active promoter	3.22 [2.34-4.43]	2.27 × 10 ^-14^	2.76 [1.99-4.03]	<5.00 × 10 ^-05^	1.15 [0.92-1.50]	1.28 × 10 ^-01^
Weak promoter	3.14 [2.31-4.27]	7.60 × 10 ^-15^	2.82 [2.09-3.98]	<5.00 × 10 ^-05^	1.21 [0.98-1.58]	4.64 × 10 ^-02^
Inactive/poised promoter	4.24 [1.84-9.77]	2.47 × 10 ^-04^	3.65 [1.76-14.19]	9.50 × 10 ^-04^	0.68 [0.40-2.00]	1.35 × 10 ^-01^
Strong enhancer (proximal)	4.18 [3.14-5.56]	6.58 × 10 ^-27^	3.93 [2.88-5.58]	<5.00 × 10 ^-05^	1.48 [1.19-1.89]	1.50 × 10 ^-04^
Strong enhancer (distal)	2.84 [2.13-3.78]	8.19 × 10 ^-14^	2.71 [2.03-3.78]	<5.00 × 10 ^-05^	1.29 [1.05-1.66]	6.35 × 10 ^-03^
Weak/poised enhancer (proximal)	2.61 [2.01-3.40]	7.69 × 10 ^-14^	2.47 [1.92-3.29]	<5.00 × 10 ^-05^	1.08 [0.89-1.32]	2.32 × 10 ^-01^
Weak/poised enhancer (distal)	2.65 [2.18-3.23]	1.14 × 10 ^-23^	2.57 [2.09-3.20]	<5.00 × 10 ^-05^	1.22 [1.06-1.42]	3.25 × 10 ^-03^
Insulator	1.76 [1.28-2.40]	4.35 × 10 ^-04^	1.69 [1.29-2.32]	1.50 × 10 ^-04^	1.06 [0.86-1.36]	3.05 × 10 ^-01^
Transcriptional transition	3.22 [2.31-4.48]	2.50 × 10 ^-13^	2.88 [2.08-4.24]	<5.00 × 10 ^-05^	1.26 [1.00-1.65]	3.25 × 10 ^-02^
Transcriptional elongation	2.45 [2.01-2.97]	1.46 × 10 ^-20^	2.13 [1.71-2.69]	<5.00 × 10 ^-05^	1.14 [1.00-1.31]	3.25 × 10 ^-02^
Weak transcribed	2.52 [2.16-2.94]	7.00 × 10 ^-34^	2.33 [1.96-2.80]	<5.00 × 10 ^-05^	1.18 [1.06-1.33]	8.50 × 10 ^-04^
Polycomb repressed	2.06 [1.67-2.52]	2.85 × 10 ^-12^	2.14 [1.66-2.78]	<5.00 × 10 ^-05^	1.31 [1.13-1.54]	1.00 × 10 ^-04^
Heterochrom; low signal	0.56 [0.48-0.64]	2.85 × 10 ^-15^	0.54 [0.45-0.64]	<5.00 × 10 ^-05^	0.94 [0.85-1.05]	1.39 × 10 ^-01^
Repetitive/CNV (proximal)	1.85 [0.49-7.04]	3.36 × 10 ^-01^	1.27 [0.54-6.02]	6.65 × 10 ^-01^	0.33 [0.18-1.00]	1.86 × 10 ^-02^
Repetitive/CNV (distal)	0.32 [0.03-2.98]	6.25 × 10 ^-01^	0.25 [0.11-Infinity]	3.02 × 10 ^-02^	0.63 [0.33-3.00]	1.46 × 10 ^-01^
DNase Clusters	2.60 [2.28-2.96]	7.63 × 10 ^-48^	2.64 [2.36-2.97]	<5.00 × 10 ^-05^	1.15 [1.06-1.26]	3.00 × 10 ^-04^
Human Insulators	2.14 [1.63-2.82]	2.19 × 10 ^-08^	1.94 [1.50-2.61]	<5.00 × 10 ^-05^	0.99 [0.83-1.23]	4.61 × 10 ^-01^

**Table 2:** Average of confidence interval width of sampling and permutations

	**Permutation**	**Sampling**
Genic and Regulatory Features	2.08	1.90
Conserved Elements	1.92	1.12
Chromatin States	2.13	1.89

The striking similarity of enrichment patterns seen overall between two independent methods provides strong evidence for the co-occurrence of trait-associated SNPs and genomic regions annotated with specific functional annotations. Of the 21 analyzed classes of annotation associated with genic features only intergenic regions showed depletion. All other genomic features associated with genes were enriched to various degrees for trait-associated SNPs (Figure 1). The odds ratio for the eQTLs (4.42 [3.52-5.54]) was the highest significant odds ratio obtained for the random sampling approach. There is growing evidence in the scientific community that eQTLs influence complex traits by measurably changing expression levels of genes [16]. The differences in significance of the odds ratios in two of the genomic features between the two methods are most likely caused by the differences of theoretical versus empirical confidence intervals (see Discussion, which highlights the value of the empirical method (circular permutations)).

### Chromatin states are a stronger predictor of trait association than sequence conservation

All of the 13 annotation classes associated with conservation and other evolutionary signatures were only modestly enriched using either method, showing odds ratios of less than two (Figure 2). Three of the annotations (evofold, vista enhancers and exapted repeats) failed to reach [19] significance. The negative set (Figure 2) is intended as an approximation to a negative control for the evolutionary signatures annotation class, and was composed of intergenic SNP data lacking any other genic or conserved/evolutionary annotations irrespective of the chromatin states present. However, almost half of trait-associated SNPs or their LD partners were found to be within this negative set (see Discussion), which could explain the modest depletion seen in that class. The relatively weak performance of evolutionary measures is a surprising result given the ubiquitous use of evolutionary conservation in computational variant prioritization approaches [20-24].

The results for 19 genomic annotations corresponding to distinct chromatin states are shown in Figure 3. Regions associated with a variety of states implicated in gene activation as identified by histone modifications were enriched for trait-associated SNPs. Strong enhancers of proximal genes (OR = 3.93 [2.88-5.58]) followed by open chromatin (OR = 3.51 [2.84-4.30]) and transcriptional transition (OR = 2.88 [2.08-4.24]) were the three most significantly enriched features identified by their distinct chromatin states for the permutation based approach. Almost three-quarters of trait-associated SNPs or their LD partners were located in regions annotated as exhibiting a relatively ‘open’, de-condensed higher order chromatin structure (Additional file 1). Strong enhancers regulating distal genes (Figure 3) were also enriched for trait-associated SNPs, albeit less so than strong enhancers which regulate proximal genes (Figure 3). Conserved distal regulatory enhancers are frequently found at loci containing developmental genes [25,26]. The results presented here may therefore reflect the depletion of variants in such enhancers due to their detrimental effects upon developmental processes. Both the relatively repressive, ‘closed’ higher order chromatin domains and heterochromatin features show depletions. Repetitive/CNV regions obtained odds ratios close to one and therefore were not significantly enriched or depleted.

Analyses were repeated using a more liberal LD cut-off point of r^2^ > 0.7 to determine LD partners. The results obtained from these data were similar to the ones obtained using r^2^ > 0.9 with only a few annotations becoming significant (Additional file 4, Additional file 5 and Additional file 6).

### Similar enrichment trends for significantly and suggestively associated SNPs

**Figure 4:**
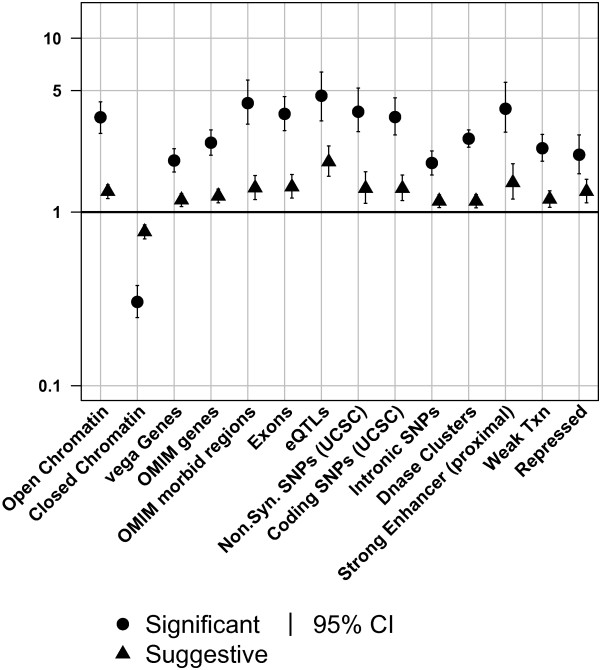
**Suggestive SNPs show modest enrichment/depletion.** Enrichment of significant and suggestive trait-associated SNPs (○,Δ) in annotations significant for suggestive trait-associated SNPs. See Additional file 2 and Table 2 for numeric values of all annotations. Solid symbols indicate odds ratios significantly different from 1 (p ≤ 0.05). Odds ratios below or above one show depletions or enrichments respectively.

There has been substantial interest in the roles of GWAS variants showing ‘suggestive’ levels of significance (i.e. SNPs with P-values = 5 × 10^-5^ – 5 × 10^-8^), as they are believed to contain many true positives with modest effect sizes [27]. If that is correct, we might expect similarities in the functional enrichment patterns of these two classes of variants. Figure 4 highlights the 14 significant enrichment/depletion results of the suggestively associated SNPs in the annotations, nine of which are from the genic annotation category. The trends are similar to those observed for genome-wide significant SNPs, but with lower odds ratios (see Additional file 7). Additional file 8 shows the results of analyses of significantly and suggestively trait-associated SNPs for all annotations.

**Figure 5:**
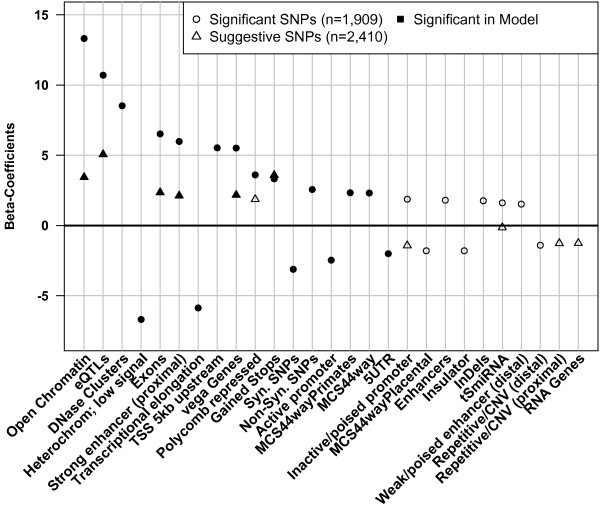
**Logistic regression identifies most influential annotations.** Significant ratio between estimated effect and standard error of annotations in the logistic regression models for the significantly and suggestively trait-associated SNPs (○,Δ) sorted after decreasing significance in the logistic regression model for significantly trait-associated SNPs. The final models for the significant and suggestive trait-associated SNPs included 27 and 12 annotations of which 17 and 6 were significant in the models, respectively. Corresponding values can be found in Table 4.

Table 3 presents the *β*-coefficient (the ratio of the estimated effect to its standard error) from the logistic regression models for the significantly and suggestively trait-associated SNPs in the final models. The significant *β*-coefficients are plotted in Figure 5. The annotations were ordered in terms of significance and effect size in the logistic regression for the significant trait-associated SNPs. Negative values implied that trait-associated SNPs were depleted in those regions, once the effects of the previously added annotations were taken into account. The final model for the significant SNPs included 25 annotations, 17 of which were significant. The OMIM morbid regions and OMIM genes were excluded from the analysis, as they were trait-associated by definition and we wanted to show the most influential annotations for trait-association status without influencing the model *a priori*. The estimated effects of the annotations ranged between −1.41 and 1.15 and the *β*-coefficient (see Methods) ranged between −6.69 and 13.31. The most significant annotations enriched for trait-associated SNPs were open higher order chromatin domains, eQTLs, DNase clusters, and exonic regions. Annotations depleted of trait-associated SNPs included the regions associated with heterochromatin and low expression signal. The high and significant enrichment signals in the open chromatin regions, eQTLs and strong enhancers (proximal) observed in the enrichment/depletion analyses were replicated in the logistic regression. Four of the annotations (transcriptional elongation, synonymous SNPs, active promoters and 5^′^UTRs) were significantly depleted in the logistic regression analysis, but showed significant enrichment when analyzed individually. Many annotations significant in the enrichment/depletion analysis when analyzed individually were not included in the final model suggesting that they have little explanatory power additional to annotations already included in the model. Annotations included in the model, such as transcriptional elongation, could have been overlooked in the enrichment/depletion analysis since their odds ratios were very similar to the rest of the annotations. However, the influence of the transcriptional elongation, when compared to the strong enhancers (proximal) in the logistic regression, is nearly equivalent in magnitude, although the direction of the weights is opposite as judged by the value of the *β* - coefficient. These differences highlight the importance of jointly analyzing all the genomic features.

**Table 3:** Comparing significant vs. suggestive logistic regression results

	**Significantly associated SNPs**				**Suggestively associated SNPs**			
**Annotation**	**Estimated effect**	**Std. error**	**β-coefficient**	**P-value**	**Estimated effect**	**Std. error**	**β-coefficient**	**P-value**
Open Chromatin	0.79	0.06	13.31	2.00 × 10^-40^	0.15	0.04	3.43	5.94 × 10^-04^
eQTLs	0.72	0.07	10.7	1.01 × 10^-26^	0.39	0.08	5.06	4.10 × 10^-07^
DNase Clusters	0.46	0.05	8.52	1.58 × 10^-17^	NA	NA	NA	NA
Heterochrom; low signal	−0.37	0.05	−6.69	2.26 × 10^-11^	NA	NA	NA	NA
Exons	0.58	0.09	6.52	6.94 × 10^-11^	0.17	0.07	2.35	1.85 × 10^-02^
Strong enhancer (A)	0.47	0.08	5.98	2.29 × 10^-09^	0.21	0.1	2.12	3.37 × 10^-02^
Transcriptional elongation	−0.43	0.07	−5.87	4.26 × 10^-09^	NA	NA	NA	NA
TSS 5 kb upstream	0.36	0.07	5.53	3.26 × 10^-08^	NA	NA	NA	NA
vega Genes	0.27	0.05	5.51	3.59 × 10^-08^	0.09	0.04	2.17	2.98 × 10^-02^
Polycomb repressed	0.24	0.07	3.6	3.19 × 10^-04^	0.13	0.07	1.86	6.32 × 10^-02^
Gained Stops	1.15	0.35	3.32	8.95 × 10^-04^	1.49	0.42	3.58	3.44 × 10^-04^
Syn. SNPs	−0.32	0.1	−3.12	1.83 × 10^-03^	NA	NA	NA	NA
Non-Syn. SNPs	0.23	0.09	2.56	1.06 × 10^-02^	NA	NA	NA	NA
Active promoter	−0.25	0.1	−2.47	1.37 × 10^-02^	NA	NA	NA	NA
MCS44wayPrimates	0.17	0.07	2.33	2.00 × 10^-02^	NA	NA	NA	NA
MCS44way	0.24	0.11	2.31	2.07 × 10^-02^	NA	NA	NA	NA
5UTR	−0.31	0.16	−2.01	4.50 × 10^-02^	NA	NA	NA	NA
Inactive/poised promoter	0.37	0.2	1.87	6.14 × 10^-02^	−0.59	0.41	−1.43	1.54 × 10^-01^
MCS44wayPlacental	−0.2	0.11	−1.8	7.14 × 10^-02^	NA	NA	NA	NA
Enhancers	0.53	0.29	1.8	7.22 × 10^-02^	NA	NA	NA	NA
Insulator	−0.18	0.1	−1.8	7.24 × 10^-02^	NA	NA	NA	NA
InDels	0.11	0.06	1.76	7.79 × 10^-02^	NA	NA	NA	NA
tSmiRNA	0.83	0.51	1.61	1.08 × 10^-01^	−9.42	66.4	−0.14	8.87 × 10^-01^
Weak/poised enhancer (B)	0.1	0.06	1.52	1.28 × 10^-01^	NA	NA	NA	NA
Repetitive/CNV (B)	−1.41	1	−1.41	1.58 × 10^-01^	NA	NA	NA	NA
Repetitive/CNV (A)	NA	NA	NA	NA	−0.9	0.71	−1.27	2.05 × 10^-01^
RNA Genes	NA	NA	NA	NA	−0.73	0.58	−1.26	2.08 × 10^-01^

Six annotations were found to have significant (P-value ≤ 0.05) effects on trait-association status of suggestive SNPs: eQTLs, open chromatin, exons, gained stop codons, vegaGenes, and strong enhancers (proximal). The annotations included in the model for suggestive SNPs had reduced *β*-coefficients when compared with their value in the logistic regression model for significantly associated SNPs, consistent with the weaker enrichments seen for the suggestive class and the mixture of true and false positives in this SNP set.

The results from logistic regression demonstrate the value of a comprehensive modeling approach that helps identify annotations providing independent information on the trait-association status of SNPs. Some of the individual annotations have highly significant effects in both the logistic regression and the enrichment analyses. Nonetheless, the overall explanatory power of the final model as evidenced from the pseudo-r^2^ values (Table 4) is relatively limited. One might imagine that the power of predictive modeling might be enhanced by the inclusion of quantitative variables rather than the essentially binary variables used here. This was borne out by an examination of models including a quantitative estimate of the upstream proximity of GWAS hits to TSSs. Additional file 9 shows two histograms of A) the upstream distance to TSS in both, significant and suggestive trait-associated SNPs, with a clear change in frequency at distances greater than 20 Kb away from the TSS and B) a closer look at the <20 Kb region. Distance to TSS was included after optimization in spite of the inclusion of other TSS associated categorical variables, such as 5 Kb upstream TSS regions. The effect of distance to TSS is negative with a *β*-coefficient of −12.18, implying that a larger distance away from the TSS results in a decrease in the likelihood that a particular SNP is significantly trait-associated. Additional file 10 shows the *β*-coefficients of the logistic regression incorporating the distance to TSS effect. A table of all coefficients is shown in Additional file 11. The pseudo-r^2^ value of the final model improved to 42% (McKelvey and Zavoina’s), relative to models lacking this variable (Table 4). Additional file 12 shows the effect of including distance to TSS for six representative genomic annotations for the significantly trait-associated SNPs. The importance of a number of other annotations declined correspondingly, with the odds ratios for strong enhancers (proximal), eQTLs and active promoters reduced the most. The reduction of the odds ratio of eQTLs confirms previous results that eQTLs are usually found close to the transcription start site of genes [28].

**Table 4:** **Pseudo-r**^**2 **^**values for logistic regressions for significantly and suggestively associated SNPs**

	**Genotyping arrays only**		**Genotyping arrays and annotations**	
	**McFadden**	**McKelvey & Zavoina’s**	**McFadden**	**McKelvey & Zavoina’s**
Significant 1 (no TSS)	0.07	0.14	0.11	0.28
Significant 2 (with TSS)	0.07	0.14	0.12	0.42
Significant 2 (without TSS)	0.07	0.14	0.11	0.28
Suggestive 1 (no TSS)	0.09	0.16	0.09	0.18
Suggestive 2 (with TSS)	0.09	0.16	0.09	0.18
Suggestive 2 (without TSS)	0.09	0.16	0.09	0.18

## Discussion

We analyzed the enrichment and depletion of significantly and suggestively trait-associated SNPs in genomic regions annotated for 58 different functional genomics features. For the significantly trait-associated SNPs we observed significant enrichment in genic annotations and several features associated with particular chromatin states. The enrichment in genic annotations has been well documented in previous studies [8,10], while there has been modest evidence for enrichment of trait-associated SNPs in regions with distinct chromatin structures [13]. However, the greatest insight is provided by logistic regression analysis, which evaluates the genomic features in terms of their influence on trait-association status in the context of the complete model for prediction of trait association status for SNPs.

Annotation classes associated with genes (Figure 1) showed enrichments and depletions comparable to previous studies. The highest significant enrichment was observed in gained stop codons obtained by the permutation method, but not the sampling method. This, and other sparsely annotated genomic features (i.e. with a low percentage of the genome covered; Additional file 1), resulted in large confidence intervals on the estimated odds ratio by either method. The observed differences in significance are due to the theoretically determined P-values, which were a widely used asymptotic approximation (see Methods), employed in the sampling method versus those determined empirically in the circular permutations method (see Methods). Theoretical values were used for the computationally intensive sampling method since they were necessarily based on a limited number of random samples, and such limitations do not apply to the permutation approach. The confidence intervals derived by permutation are generally slightly more conservative (i.e. larger) than those from the sampling approach. This is consistent with the permutation approach taking appropriate account of non-random distributions of annotations and SNP locations. As expected among the genomic features enriched for significantly trait-associated SNPs were the OMIM morbid regions, identified as regions associated with traits in GWAS and linkage studies [29]; these regions may approximate a positive control.

Conserved elements and regions with other evolutionary signatures (Figure 2) usually exhibited significant though modest enrichment with odds ratios ranging from 1.64 to 1.97. Odds ratios for evofold, VISTA enhancers and exapted repeats were found to be not significant, but other conserved regions and evolutionary signatures showed odds ratios comparable to each other. The PREMOD [30] annotation is the only annotation obtained from a predictive algorithm that shows significant enrichment (OR = 1.64 [1.38-1.97]). It is, unlike other algorithms, not restricted to modules located proximal to genes, but mostly contains distal predicted *cis*-regulated module predictions [30]. This has implications for follow-up studies, as trait-associated SNPs in conserved regions tend to be prioritized for further studies [31] either manually or via algorithms [20,21,23,32]. Conserved regions have also been shown to add little information to the prediction of nucleotides acting as eQTLs, with no significant odds ratios of enrichment observed for conserved phastCons elements [33]. It is likely that many of the most conserved sites (‘MCS’, obtained through phastCons) are shared among the sets of sites identified using different alignments with a varying number of investigated species (Figure 2).

We detected enrichment for trait-associated SNPs in various chromatin states (Figure 3) associated with a variety of regulatory functions. The proximal and distal sets of the chromatin states influence expression of proximal and rather more distant distal genes, respectively [13]. The significant enrichment signal in the enhancer annotation is consistent with the results of Ernst et al. [13], who investigated GWAS results for immune and blood related phenotypes in chromatin data from the GM12878 lymphoblastic cell line. The authors reported a two-fold enrichment for a combination of the proximal and distal strong enhancers for SNPs associated with leukemia, rheumatoid arthritis, and systemic lupus erythematosus [13]. We are able to confirm their observed enrichment of trait-associated SNPs and also observe enrichment signals with larger odds ratios in the strong enhancer sets of proximal genes than for the strong enhancer set of distal genes. A third of the significantly trait-associated SNPs used here are associated to immunity-related traits, so a strong signal in the enhancer regions of a lymphoblastic cell line is intuitively reasonable. A clear difference is seen between open and closed domains of higher order chromatin. This was expected as closed chromatin is known to contain a somewhat higher density of SNPs [15], but is also likely to contain fewer trait-associated SNPs due to low gene density [17]. Open chromatin, however, is present at gene and regulatory feature dense areas [17] and is therefore more likely to harbor trait-associated variants.

The suggestively trait-associated SNPs showed similar results to the significantly associated SNPs, but with more moderate odds ratios. This result is consistent with suggestively associated SNPs containing both false positives (which we would expect to have no bias towards particular annotations) and true associations, whose effects were not of sufficient magnitude to show genome wide significance. These true positives would be expected to have the same bias towards particular genomic features as trait-associated SNPs of genome wide significance [10].

While significantly trait-associated SNPs are consistently documented, suggestive associations often remain unreported, since they are assumed to contribute less to our understanding of the underlying biology. Additionally, the NHGRI GWAS catalog only incorporates SNPs with association levels starting at 5 × 10^-5^, where the more commonly accepted level for suggestively associated SNPs starts at 5 × 10^-4^[5,7]. This means that the significantly associated SNP set is likely to be a more comprehensive and complete SNP set, despite containing a smaller number of SNPs. The similarity of enrichment trends between the significant and suggestive sets are encouraging and may be of use to aid further research into areas of the human genome surrounding suggestively trait-associated variants on exonic variants, which may introduce bias towards certain genomic features, such as genic regions, which may affect the results for the suggestively associated SNPs more than the significantly associated SNPs, as there are more of the latter. However, the enrichment trends between the two sets suggest that this is not a major problem. The results are therefore encouraging and may be of use to aid further research into areas of the human genome surrounding suggestively trait-associated variants.

A combination of the two full logistic regression models identified six annotations that significantly influenced trait-association status for both significantly and suggestively associated SNPs. These annotations were open chromatin, eQTLs, exons, strong enhancers (proximal) of proximal genes, vegaGenes, and gained stop codons. These results are biologically reasonable, as the disruption of coding regions of genes gives rise to different phenotypes. Open chromatin is, as mentioned before, densely populated by genes and regulatory features, while recent literature indicates that eQTLs are highly influential in causing phenotypic variation by regulating gene expression [16,28,34-37]. In the significantly trait-associated model, the conserved regions included were the most conserved elements identified in the primate lineage, followed by all conserved sites identified between 44 vertebrates. This suggests that these two levels of conservation are sufficiently different from each other to be separately included in the model. The previously observed trend of more moderate effects in the suggestively trait-associated SNP dataset was confirmed in all genome annotations, with the exception of the gained stop codons, which had a stronger effect on suggestively associated SNPs.

The majority of significantly trait-associated SNPs and their LD partners (55%) overlap in regions identified as containing genes listed in the vega database [29,38]. This percentage can be increased to 70% by adding the remaining 7 genic annotations found to influence significant trait-association status: eQTLs, exons, TSS 5 Kb upstream, gained stops, synonymous SNPs, non-synonymous SNPs and 5^′^UTR. One or more of the conserved region annotations overlap with 72% of significantly trait-associated SNPs, which is reduced to 48% if the genomic features overlapping with the highest number of SNPs, positively selected genes and regions showing constraint in the accumulation of indels, are excluded from that analysis. These latter two genomic features contained the highest number of trait-associated SNPs in regions with evolutionary signatures. Most widely used prediction algorithms [20,32] already make use of conserved sites to predict trait-associated SNPs, but could possibly be improved if conserved indels were included into their predictive methods. The negative set was overlapping with 47% of the trait-associated SNPs. It is, for example, possible that a SNP overlapping with conserved sites has LD partners, which overlap with the negative sequence annotation.

The variables identified as informative in the logistic regression do indeed harbor many trait-associated SNPs on closer inspection. Some (4%) significantly trait-associated SNPs or their LD partners overlapped with none of the genomic features with a positive influence on trait-association status. In contrast, 23% of background SNPs were not overlapping any of those genomic features (Additional file 13). The odds ratio, which was calculated for the observed distribution of significantly associated SNPs and their LD partners that are overlapping with the 12 identified annotations with a positive *β*-coefficient, was 7.70 [6.09-9.73] and a P-value of 6.19 × 10^-126^. The 4.2% of the trait-associated SNPs that are not explained by those 12 annotations overlap mainly with heterochromatin and intergenic regions. The chromatin states defined by Ernst et al. [13] cover the entire genome, so that all trait-associated SNPs co-occur with at least one of the states.

Table 4 shows the McFadden’s and McKelvey and Zavoina pseudo-r^2^ values for the empty and full models with and without distance to TSS for suggestively and significantly trait-associated SNPs. The logistic regression model without the distance to TSS for the significantly associated SNPs explained 11-25% of the observed variance, which was an increase of 4-11% when compared to the empty model, which only included the effects of the genotyping arrays. An ANOVA test, using a chi-squared test, showed the difference between the two models to be significant (Deviance = 1501.00, P-value = 3.13 × 10^-309^). The difference between the two models for the suggestively associated SNPs was also significant, albeit less so (Deviance = 113.06, P-value = 1.49 × 10^-18^). The pseudo-r^2^ values for the model including the distance to TSS range from 12-42% depending on the method to calculate the value. Although the full model without the Distance to TSS variable is a substantial improvement on the empty model, the pseudo-r^2^ suggests much variance remains unexplained. This is hardly surprising when it is considered that the data contains millions of SNPs which are functionally annotated, either directly or through LD partners, but which are not known to be trait-associated. This indicates that there is quite some way to go before one can use annotation information to predict trait-association status with any confidence. Improvements in the accuracy and precision of annotation will undoubtedly help – for example the resolution of conserved regions or chromatin states can be expected to improve over time. Such improvement combined with better information on SNP trait association status such as effect size or size and power of individual studies might further speed progress towards models that are better able to predict functionally relevant SNPs, either for focused functional studies, or for inclusion in health prediction algorithms. Additionally, the investigation using distance to TSS highlights the importance of quantitative variables, which may be a future avenue to take, once these annotations become available.

## Conclusion

We have identified genomic features which are significantly enriched or depleted for both significantly and suggestively trait-associated SNPs. Additionally, we were successful in assigning weights to 17 genomic features, which indicate their relative influence on trait-association status of GWAS hits significant at the genome-wide level. These weights could be used to further prioritize GWAS hits as candidates for potential follow-up studies. The most informative and influential genomic features for significant trait-association status were regions associated with particular chromatin states, as identified using logistic regression. Conserved elements and regions with other evolutionary signatures were shown to have relatively weaker influence than either chromatin states or genic region annotations, once all other included genomic features were taken into account. Distance to transcription start site (TSS) was identified as an influential factor, where SNPs further away from the TSS were less likely to be significantly trait-associated. We have also identified four genomic features – synonymous SNPs, transcriptional elongation, 5^′^UTRs and active promoters – that are enriched for significantly trait-associated SNPs in both the circular permutations and sampling method, but show relative depletion in the logistic regression model, which looks at relative influences across the analyzed genomic features. This stresses the value of studying combined influences of the genomic features relative to each other, rather than separately. With the data in place, we can now investigate different trait-subsets and other co-occurrences within the genome.

## Methods

### Trait-associated SNPs

The significantly and suggestively trait-associated SNP sets were derived from the NHGRI GWAS catalogue; accessed 25 Aug 2011 [5]. This dataset reported 4,520 SNPs with at least one associated trait (5,800 reported associations in total) from 764 studies. A unique SNP is the “rs” number of a SNP that is associated to at least one trait. The common genome-wide level significance threshold (*p* < 5 × 10^-8^) was used to define 1,909 significantly trait-associated SNPs from 586 studies. The suggestively trait-associated SNPs set was defined as SNPs with association P-values between 5 × 10^-8^ and 5 × 10^-5^. SNPs that were located on either the Y-chromosome or unassigned chromosomes were removed from all analyses. SNPs in the suggestively associated SNPs set found to be in LD (r^2^ > 0.9) with significant SNPs were removed from the dataset, resulting in 2,410 unique rs numbers from 412 studies.

### Total number of analyzed SNPs

A list of 3,840,944 SNPs incorporated all SNPs that were included on different genotyping arrays and also those that are part of the HapMap CEU II data. The latter was included to account for SNPs that were identified as trait-associated SNPs through imputation in meta-analyses. The list included information on linkage disequilibrium (LD) partners of all SNPs (see below), the location of SNPs in the genome and the observed co-occurrences with annotations for the selected genomic features (see below). Autosomes and the X chromosome were analyzed in this study.

### LD partners

The HapMap CEU II data was used to define LD partners of all SNPs. LD partners were defined as SNPs from the total set in LD (r^2^ > 0.9) with the analyzed SNPs [39,40]. The distance between LD partners was up to 250 Kb on either side of the SNP. The maximum distance between two LD partners for any particular SNP was therefore up to 500 Kb. This r^2^ was chosen since the effect of SNPs in LD of that value are said to be equivalent in trait-association studies [8].

### Genomic features

This study analyzed three main categories of annotations: genic and regulatory features, regions with conserved or evolutionary signatures, and chromatin states. Additional file 14 provides further details of the annotations and their sources. All annotations were downloaded in hg18, where available. If they were not available, the UCSC liftOver tool was used to transfer the annotated regions into hg18 [41]. A SNP was annotated as overlapping within a particular genomic feature if it or any of its LD partners was located within the annotation. Trait-associated SNPs without LD partners were analyzed on their own. We also included a derived annotation as an approximation for a negative control: From the intergenic dataset we excluded sites overlapping with any form of evolutionary, regulatory or genic annotation examined here, so that it is negative for sequence annotation irrespective of epigenetic state.

### Odds ratios

For a particular genomic feature, one overlap was counted, if a trait-associated SNP or any of its LD partners co-occurred in a region annotated for that genomic feature. Non-overlaps were defined as the lack of co-occurrences between a trait-associated SNP or its LD partners and an annotation. Odds ratios were calculated to enable comparisons with previous studies [8], where an odds ratio was defined as the product of the overlaps of the real data and the non-overlaps of the sample data divided by the product of the non-overlaps of the real data and the overlaps of the sample data, i.e.: [(Overlaps Real Data)* (Non-Overlaps Sample Data)]/[(Non-Overlaps Real Data)* (Overlaps Sample Data)].

Odds ratios of enrichment/depletion were calculated by comparing overlaps between genomic features and real trait-associated data with overlaps of SNPs determined by chance alone. The ‘epitools’ package [42] of the statistical program R version 2.12.1 [43] was used for the calculations. Odds ratio P-values were significant when below the Bonferroni-corrected significance threshold, which in our case was calculated for 58 independent variables (P ≤ 8.62 × 10^-4^). These annotations were not independent from each other, which means that the Bonferroni corrected P-value is conservative. Fold enrichment, a ratio of hits in the associated data over the hits in the permuted data, was calculated for the significant SNP set to compare it to the calculated odds ratio to aid the interpretation of results.

### Sampling genotyping SNP platforms

The sampling method was based on Hindorff et al. [8] and aimed to obtain sample sets of SNPs of equal size to the set of trait-associated SNPs represented on genotyping platforms. We used weighted groups based on the manufacturer(s) of the SNP platform(s) to draw the samples, rather than on individual genotyping arrays, as that information was often unavailable. The numbers of SNPs drawn from each manufacturer group were proportional to the number of SNPs observed in the real data. The HapMap CEU II SNPs were included to account for the trait-associated SNPs obtained from GWAS using imputed genotypes. The LD partners were ascertained as for the trait-associated SNPs. Odds ratios, confidence intervals and P-values indicating the significance of the observed results were calculated using the oddsratio.wald() function from the ‘epitools’ R package. This function calculated the odds ratios by comparing unconditional maximum likelihoods of the observed value compared with the mean number of hits of the 100 samples.

### Chromosome-bound circular permutations

A novel permutation approach was applied, which preserved the internal structure of the datasets in terms of relative distance between SNPs, the observed clustering of annotations, and the LD structure around SNPs. GWAS hit status (appears in NHGRI GWAS catalogue with required p-value or not) was established for a list of a total of 3,840,944 known HapMap CEU II SNPs in autosomes and the X chromosome with information on LD (r^2^ > 0.9) SNP partners for each SNP appearing on the list. For each permutation a randomly generated number, drawn from a uniform distribution between one and the number of SNPs per analyzed chromosome, was used to shift the trait-association status of all SNPs within a chromosome. Permutations were circular within chromosomes: where a shift of status exceeded the SNPs available before the end of the chromosome it resumed at the beginning of the chromosome. This produced a population of 20,000 permuted genomes containing the same number of trait-associated SNPs and showing the same degree of genomic clustering as observed in the original SNP datasets. Overlaps between the permuted trait-associated variants and the annotations were counted. The odds ratios were calculated by comparing the mean number of overlaps of permuted SNPs and the observed results for the associated SNPs for each annotation. The 95% confidence intervals were obtained by calculating odds ratios for the 5th and 95th largest values of the permuted hits. The P-value of the odds ratios obtained by the permutations was calculated from the proportion of permuted datasets that were more extreme than the observations in the real trait-associated SNP set. Hence the lower bound of the P-value was 5 × 10^-5^ when results from the real data were more extreme than any of the 20,000 permutations.

### Logistic regression to establish relative importance of annotations

Logistic regression was applied in order to model annotations as variables that influenced trait-association status of SNPs. The trait-association status was modeled as a binary variable, where trait-associated SNPs were coded as one and non-associated SNPs were coded as zero. The analysis used the established SNP set with 3,840,944 SNPs and regressed the explanatory variables (co-occurs with annotation or not) onto the trait-association status at the chosen significance threshold. The genotyping SNP platforms and HapMap CEU II SNPs were included as explanatory variables in the starting or empty model, as presence on a SNP genotyping array was significant in explaining trait-association status in previous analyses. A stepwise logistic regression determined the ex- or inclusion of annotations in the model based on a reduction of the Akaike’s Information Criterion (AIC) to (‘MASS’ package [44] for R). The process halted, when the AIC increased rather than decreased with additional variables. An estimated effect on trait-association status, its standard error and P-value were extracted for each included variable from the summary of the final model. A *β*-coefficient, the ratio of estimated effect and its standard error, was calculated. The magnitude of the *β*-coefficients indicates the trend and magnitude of the effect of a variable, here of an annotation. The P-value associated to the *β*-coefficients indicates the significance of the annotation within the model.

### Calculations of pseudo-r^2^ values

One interpretation of the r^2^ goodness-of-fit parameter derived from linear regression is as the proportion of the data variation that the model explains. Logistic regression models do not generate an r^2^ value as such. However, a pseudo r^2^ can be estimated in a variety of ways. Here, we chose the McFadden’s pseudo-r^2^ as well as McKelvey and Zavoina’s pseudo-r^2^. McFadden’s pseudo-r^2^ is calculated by the ratio of the log likelihoods from empty and full models [45]. McKelvey & Zavoina’s pseudo-r^2^ is calculated using predicted values of the dependent variable [45]. The R package ‘descr’ (function LogRegR2) [46] was used to calculate the pseudo-r^2^ values.

## Competing interests

The authors declare that they have no competing interests.

## Authors’ contributions

ASK carried out the statistical analyses and drafted the manuscript. PN, CAMS and CSH conceived of the study, supervised analyses and helped draft the manuscript. All authors read and approved the final manuscript.

## Supplementary Material

Additional file 1 **Statistics of genomic features.** Containing distribution statistics of the analyzed genomic features including number of sites, percentages of nucleotides covered, percentages of SNPs covered and the average length of the annotated nucleotide sites. Click here for file

Additional file 2 **Fold Enrichment for significantly trait-associated SNPs.** Comparing odds ratios obtained by permutations, fold enrichment and obtained percentages for observed and permuted significantly trait-associated SNPs. Click here for file

Additional file 3 **Fold enrichment and odds ratios.** Showing that Odds ratios and fold enrichment are strongly correlated with each other (r^2^ = 0.96). The correlation is highly significant (P-value = 7.8 × 10^-40^) indicating that odds ratios can be interpreted as fold enrichment. Click here for file

Additional file 4 **Different r**^**2 **^**threshold comparisons in genic regions.** Showing the comparison between two different r^2^ thresholds for the genic regions. Overall the odds ratios do not show significant differences. Click here for file

Additional file 5 **Different r**^**2 **^**threshold comparisons in regions of conserved and evolutionary signatures.** Showing the comparison between two different r^2^ thresholds for the conserved regions. Overall the odds ratios do not show significant differences. Click here for file

Additional file 6 **Different r**^**2 **^**threshold comparisons in regions associated with different chromatin states.** Showing the comparison between two different r^2^ thresholds for the different chromatin states. Overall the odds ratios do not show significant differences. Click here for file

Additional file 7 **Correlation between ratio of odds ratios and CI width for significantly and suggestively trait-associated SNPs.** Between the ratios of odds ratio and confidence interval width of significant and suggestive SNPs (r^2^ = 0.89; P-value = 1.60 × 10^-26^). The ratio can be a lot higher for suggestive SNPs, indicating that the confidence intervals are shorter. However, this may be due to a difference in the number of analyzed SNPs between the two datasets. Click here for file

Additional file 8 **Comparison of permutation results of significantly and suggestively trait-associated SNPs.** That compares all permutation results for significantly and suggestively trait-associated SNPs. Click here for file

Additional file 9 **Histogram of distance to TSS for significantly trait-associated SNPs.** Showing the distribution of distance to TSS across all significant and suggestive trait-associated SNPs and a close up of the <20 Kb region. Click here for file

Additional file 10 **Distance to TSS logistic regression graph.** Showing the new *β*-coefficients of the genomic annotations in the logistic regression analysis. Click here for file

Additional file 11 **Table of distance to TSS log reg.** Showing the estimated effect, standard error of the estimated effect, β-coefficient and the P-value of the genomic annotation included in the optimized model incorporating the distance to TSS variable. Click here for file

Additional file 12 **Table of changing odds ratios of select annotations before and after taking distance to TSS into account.** Showing the changes in odds ratios of six different annotations before and after distance to TSS was included into a logistic regression model. Click here for file

Additional file 13 **Histogram of annotation overlaps for SNPs.** Representing the percentage of SNPs (y-axis) overlapping with different numbers of annotations (x-axis) in all SNPs (grey) and the significantly trait-associated SNPs (black). A) Histogram of annotations identified to have a positive *β*-coefficient. For all SNPs: Mean: 1.89, Standard Deviation: 1.83. Trait-associated SNPs: Mean: 3.60, Standard Deviation: 2.26. B) Histogram of annotations identified to have a negative *β*-coefficient. For all SNPs: Mean: 0.54, Standard Deviation: 0.98. Trait-associated SNPs: Mean: 1.07, Standard Deviation: 0.78. Click here for file

Additional file 14 **Sources and description of genomic features.** Containing a full description of all analyzed genomic features and their online sources. Click here for file
