# Supplementary figures and images for: The genomic signature of trait-associated variants
Source: BMC Genomics. 2013 Feb 18;14:108. doi: 10.1186/1471-2164-14-108 (PMC3600003; doi:10.1186/1471-2164-14-108)

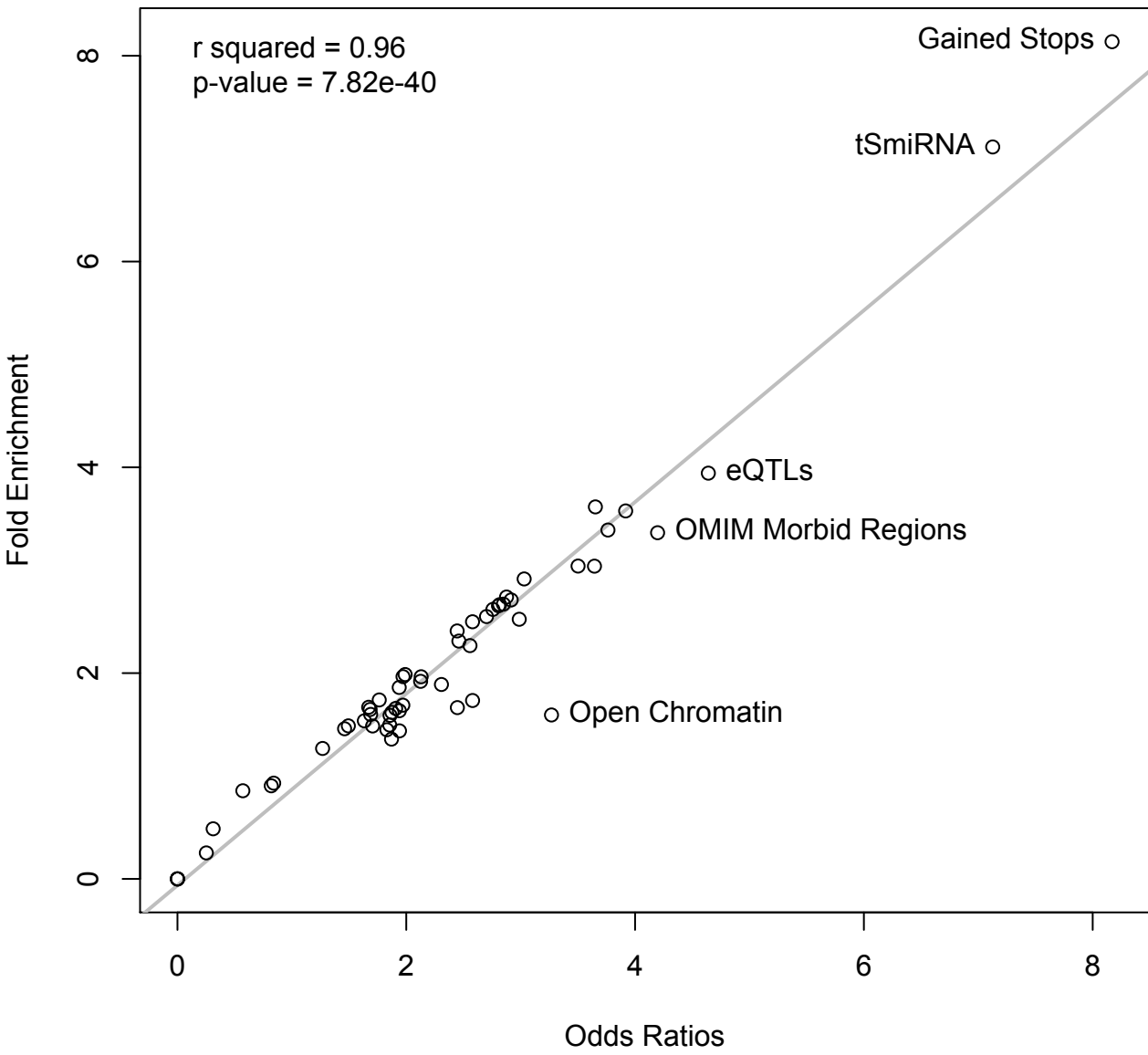

Supplement: Additional file 3 — Fold enrichment and odds ratios. Showing that Odds ratios and fold enrichment are strongly correlated with each other (r2 = 0.96). The correlation is highly significant (P-value = 7.8 × 10^-40^) indicating that odds ratios can be interpreted as fold enrichment. [file 1471-2164-14-108-S3.pdf]

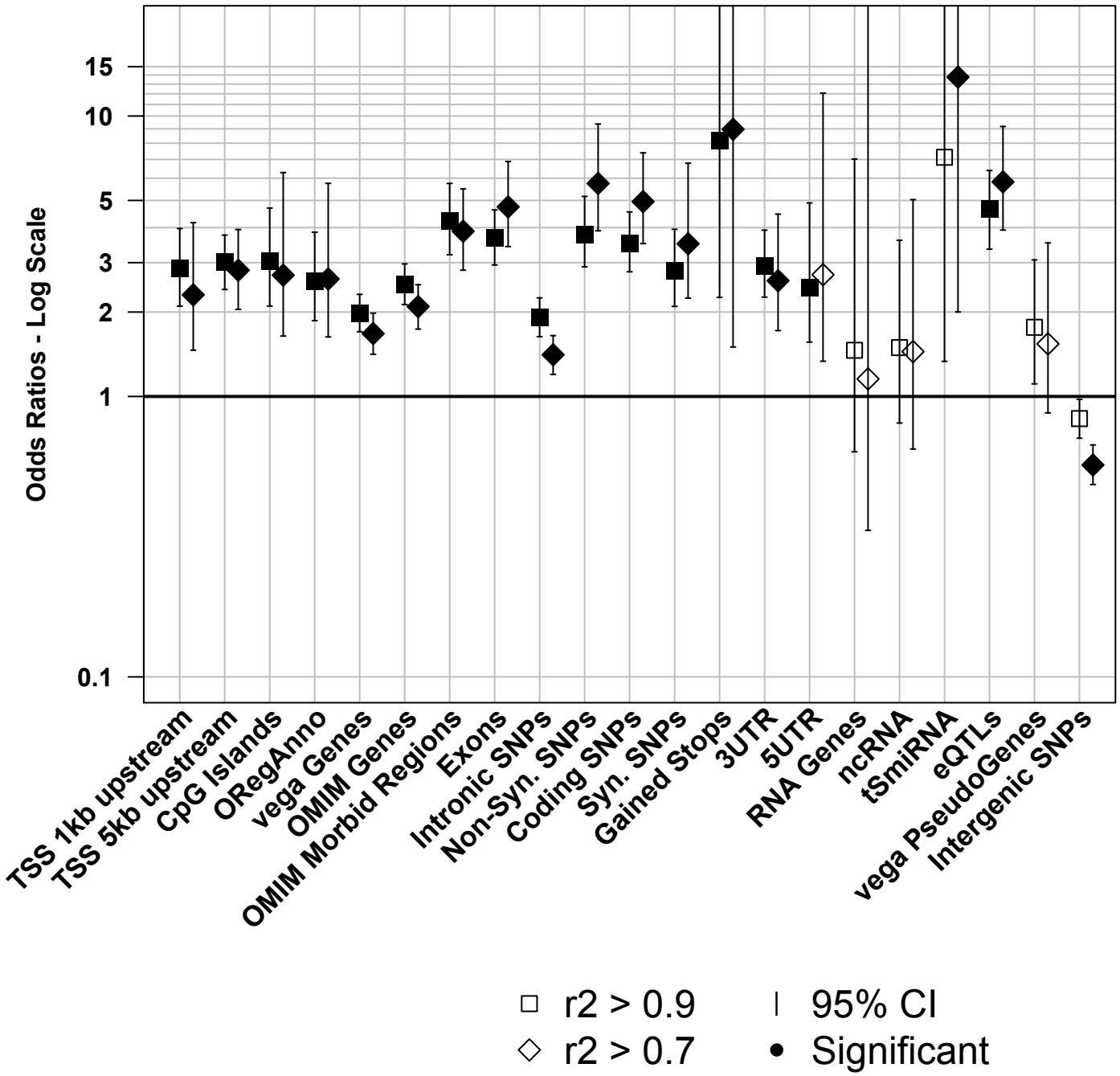

Supplement: Additional file 4 — Different r2 threshold comparisons in genic regions. Showing the comparison between two different r2 thresholds for the genic regions. Overall the odds ratios do not show significant differences. [file 1471-2164-14-108-S4.pdf]

Odds Ratios - Log Scale

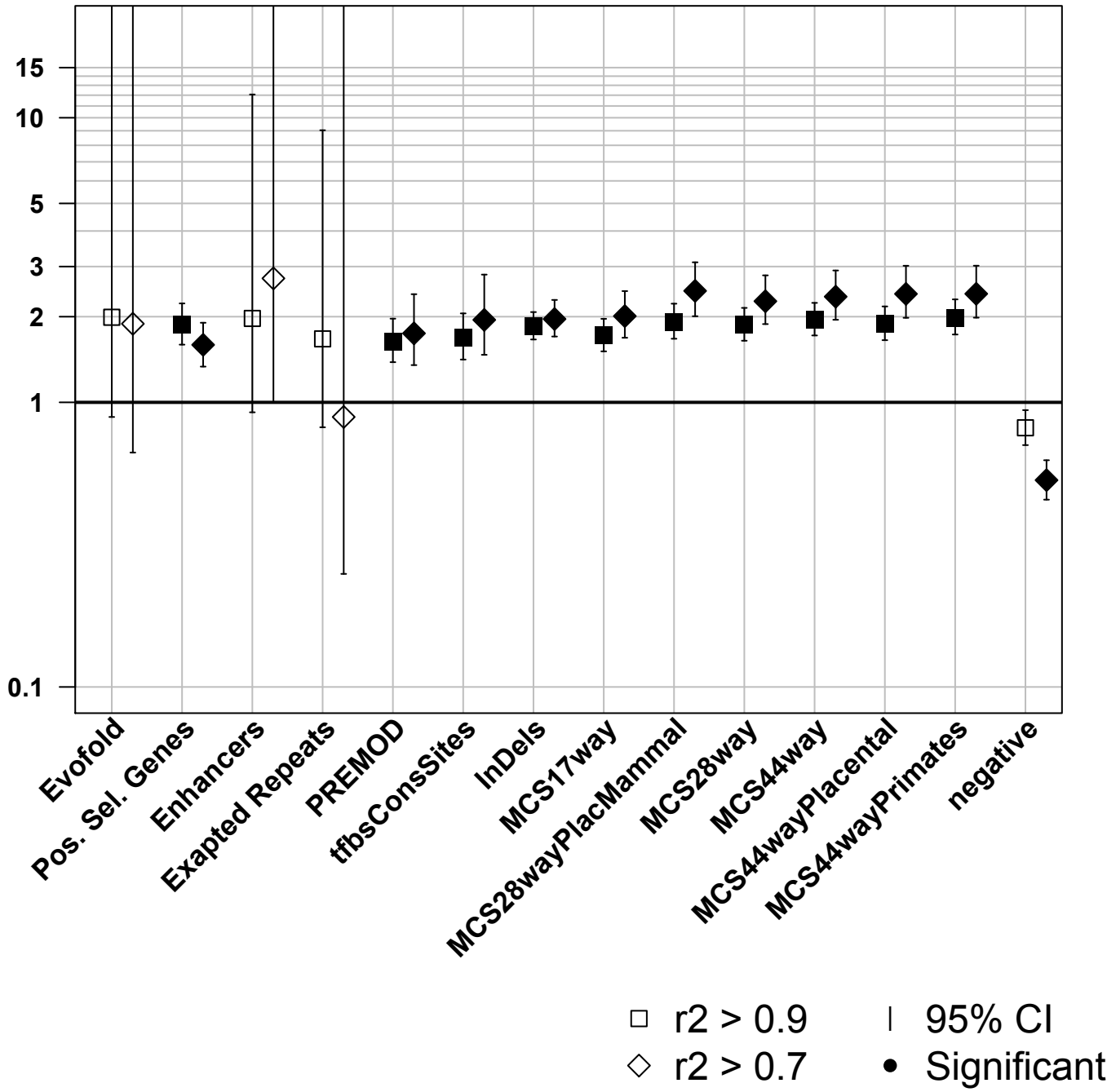

Supplement: Additional file 5 — Different r2 threshold comparisons in regions of conserved and evolutionary signatures. Showing the comparison between two different r2 thresholds for the conserved regions. Overall the odds ratios do not show significant differences. [file 1471-2164-14-108-S5.pdf]

Odds Ratios - Log Scale

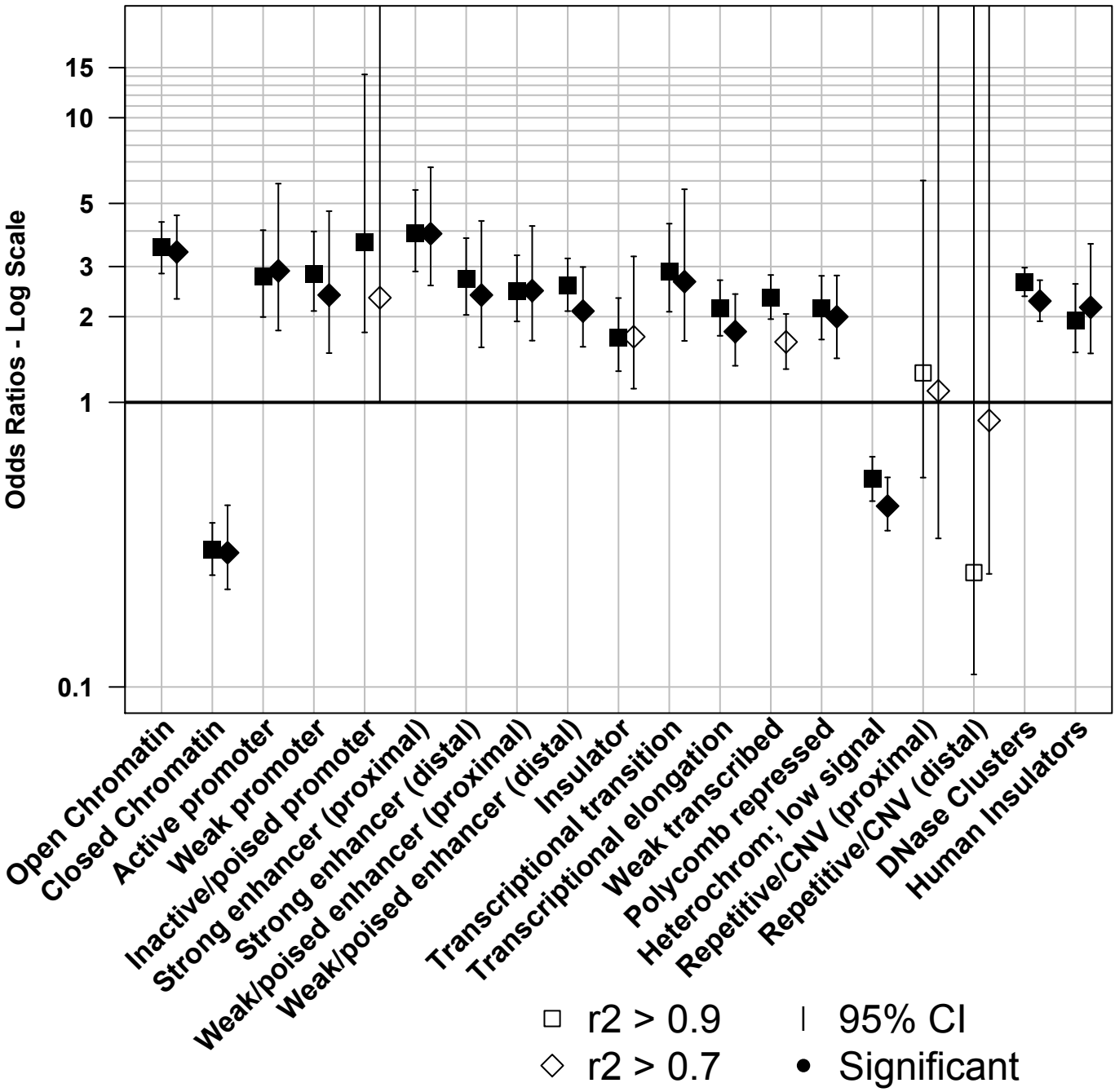

Supplement: Additional file 6 — Different r2 threshold comparisons in regions associated with different chromatin states. Showing the comparison between two different r2 thresholds for the different chromatin states. Overall the odds ratios do not show significant differences. [file 1471-2164-14-108-S6.pdf]

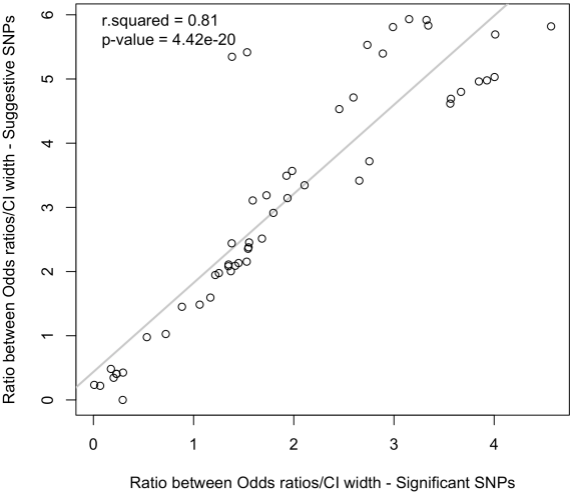

Supplement: Additional file 7 — Correlation between ratio of odds ratios and CI width for significantly and suggestively trait-associated SNPs. Between the ratios of odds ratio and confidence interval width of significant and suggestive SNPs (r2 = 0.89; P-value = 1.60 × 10^-26^). The ratio can be a lot higher for suggestive SNPs, indicating that the confidence intervals are shorter. However, this may be due to a difference in the number of analyzed SNPs between the two datasets. [file 1471-2164-14-108-S7.pdf]

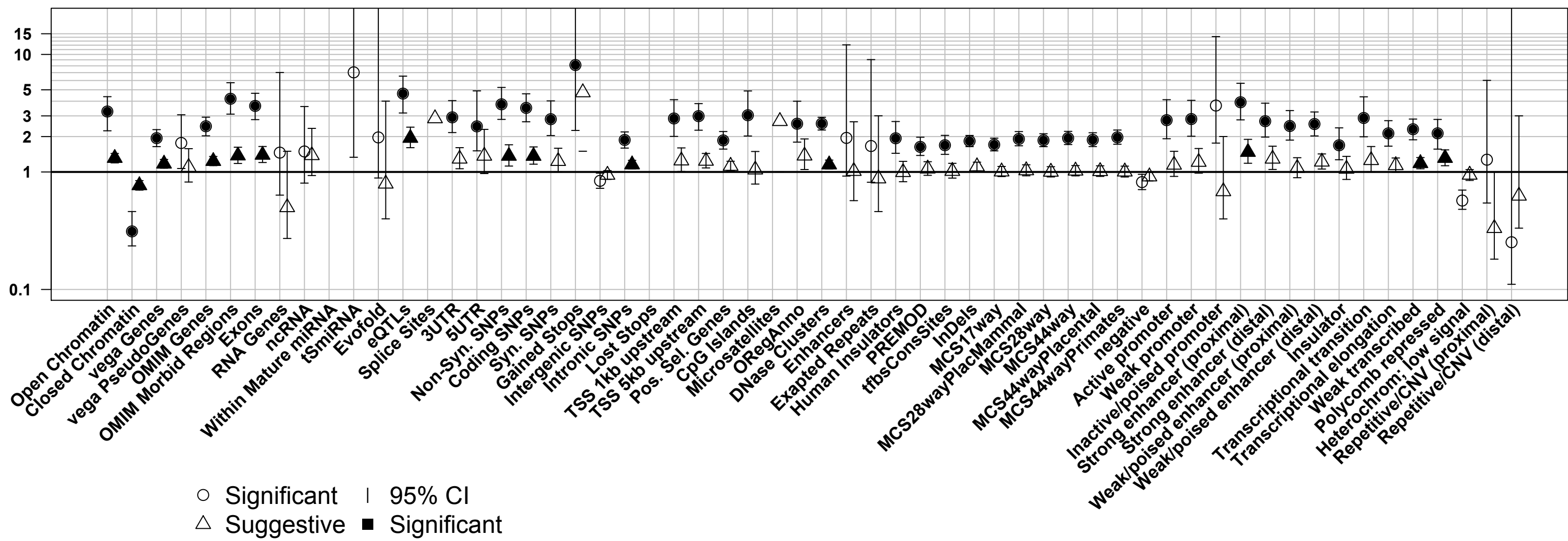

Supplement: Additional file 8 — Comparison of permutation results of significantly and suggestively trait-associated SNPs. That compares all permutation results for significantly and suggestively trait-associated SNPs. [file 1471-2164-14-108-S8.pdf]

**A**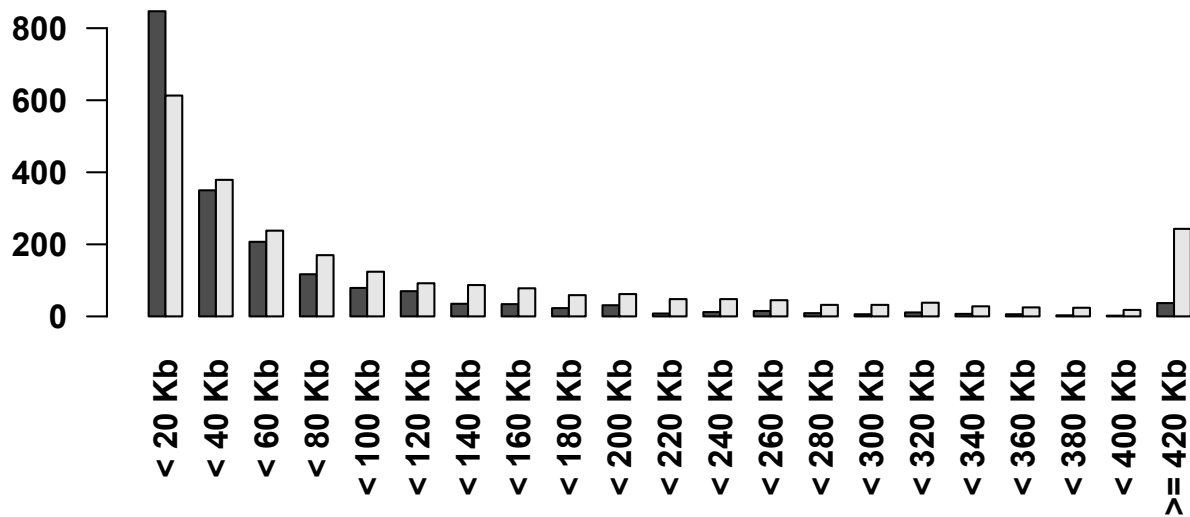**B**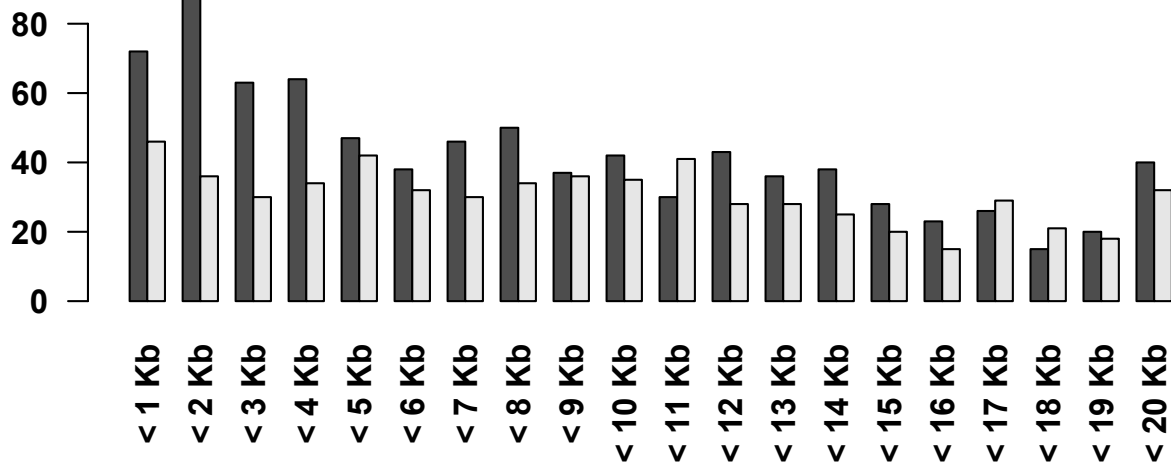

Supplement: Additional file 9 — Histogram of distance to TSS for significantly trait-associated SNPs. Showing the distribution of distance to TSS across all significant and suggestive trait-associated SNPs and a close up of the <20 Kb region. [file 1471-2164-14-108-S9.pdf]

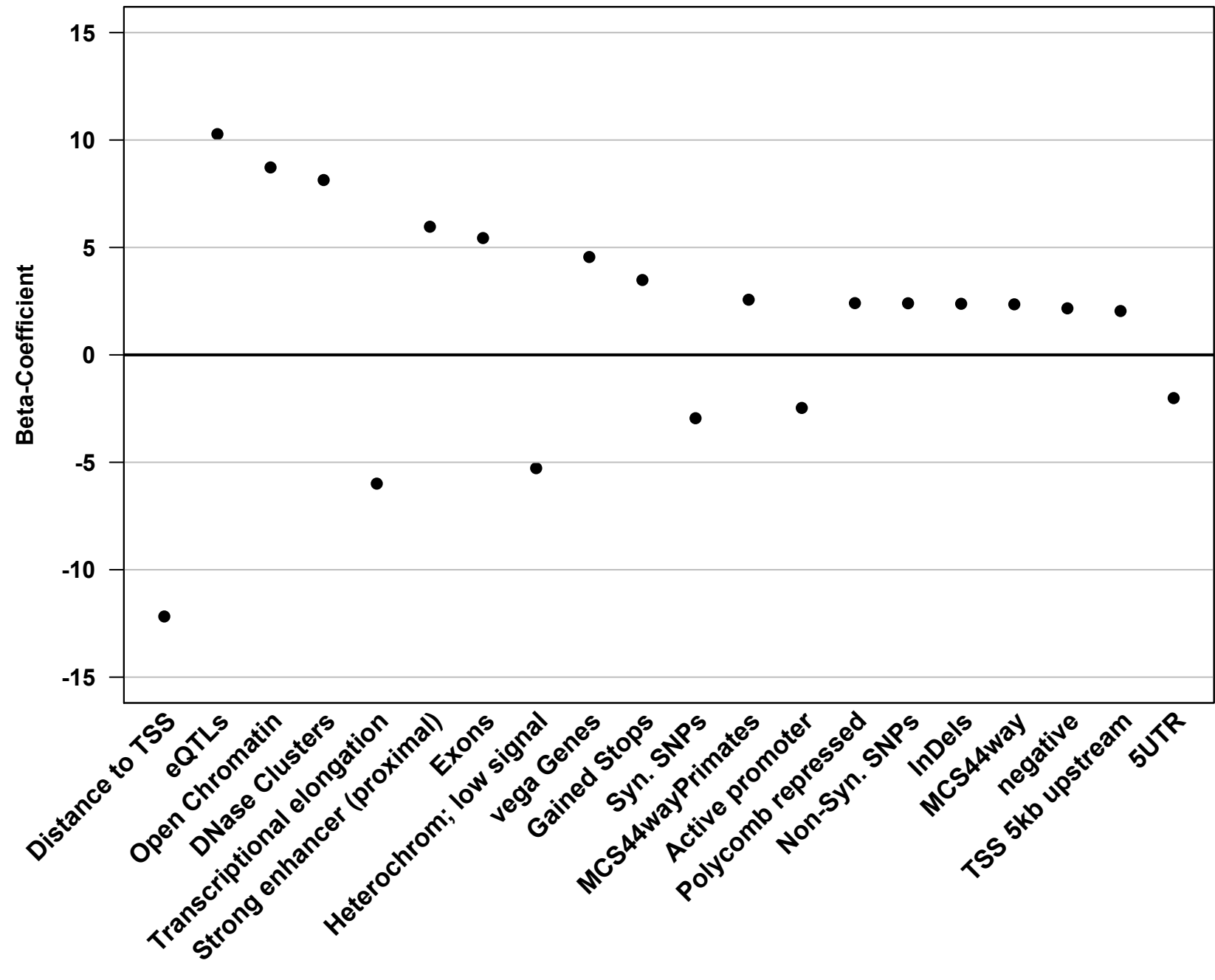

Supplement: Additional file 10 — Distance to TSS logistic regression graph. Showing the new β-coefficients of the genomic annotations in the logistic regression analysis. [file 1471-2164-14-108-S10.pdf]

A

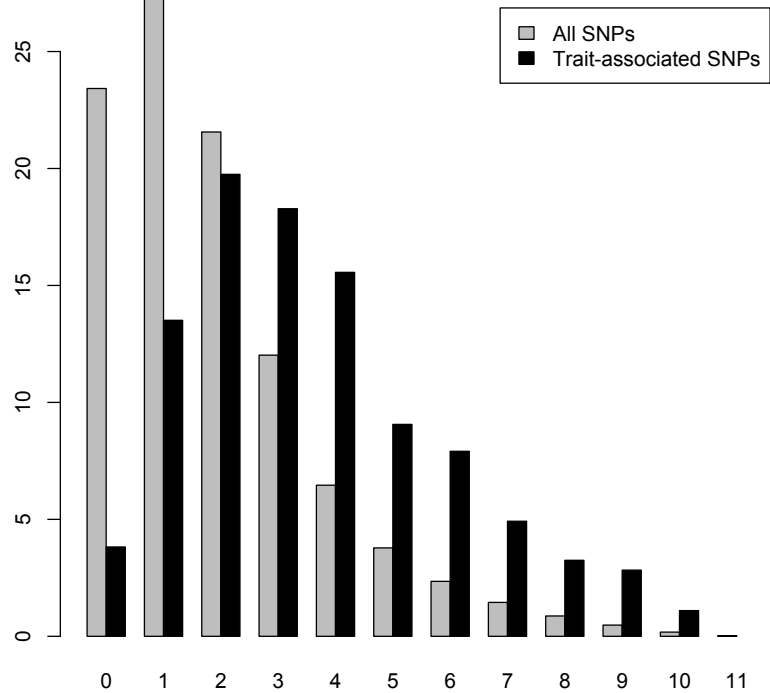

B

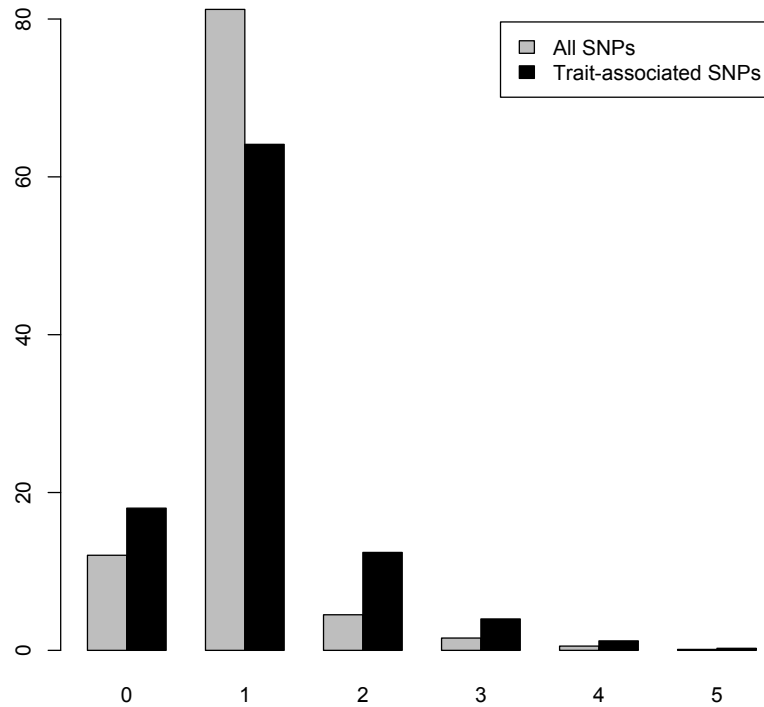

Supplement: Additional file 13 — Histogram of annotation overlaps for SNPs. Representing the percentage of SNPs (y-axis) overlapping with different numbers of annotations (x-axis) in all SNPs (grey) and the significantly trait-associated SNPs (black). A) Histogram of annotations identified to have a positive β-coefficient. For all SNPs: Mean: 1.89, Standard Deviation: 1.83. Trait-associated SNPs: Mean: 3.60, Standard Deviation: 2.26. B) Histogram of annotations identified to have a negative β-coefficient. For all SNPs: Mean: 0.54, Standard Deviation: 0.98. Trait-associated SNPs: Mean: 1.07, Standard Deviation: 0.78. [file 1471-2164-14-108-S13.pdf]
